# Using the affective bias test to predict drug‐induced negative affect: implications for drug safety

**DOI:** 10.1111/bph.13972

**Published:** 2017-08-30

**Authors:** S A Stuart, C M Wood, E S J Robinson

**Affiliations:** ^1^ School of Physiology and Pharmacology, Biomedical Sciences, University Walk University of Bristol Bristol UK

## Abstract

**Background and Purpose:**

Predicting the risk of drug‐induced adverse psychiatric effects is important but currently not possible in non‐human species. We investigated whether the affective bias test (ABT) could provide a preclinical method with translational and predictive validity.

**Experimental Approach:**

The ABT is a bowl‐digging task, which quantifies biases associated with learning and memory. Rats encounter independent learning experiences, on separate days, under either acute manipulations (e.g. pro‐depressant vs. control) or different absolute reward values (e.g. high vs. low). A bias is observed during a preference test when an animal's choices reflect their prior experience. We investigated the effects of putative pro‐depressant drug treatments following acute or chronic administration on the formation of an affective bias or reward‐induced positive bias respectively.

**Key Results:**

The immunomodulators LPS (10 μg·kg^−1^), corticosterone (10 and 30 mg·kg^−1^) and IFN‐α (100 U·kg^−1^) induced a negative affective bias following acute treatment. Tetrabenazine (1 mg·kg^−1^) also induced a negative bias, but no effects were observed with varenicline, carbamazepine or montelukast. Chronic treatment with IFN‐α (100 U·kg^−1^) and retinoic acid (10 mg·kg^−1^) impaired the formation of a reward‐induced positive bias but did not alter sucrose preference test (SPT).

**Conclusions and Implications:**

The ABT has the potential to provide a novel approach to predict pro‐depressant risk in a non‐human species. Negative biases induced by acute treatment in the standard version of the task may also predict longer‐term effects on reward processing as shown by the deficit in reward‐induced positive bias following chronic treatment, an effect distinct from anhedonia in the SPT.

**Linked Articles:**

This article is part of a themed section on Pharmacology of Cognition: a Panacea for Neuropsychiatric Disease? To view the other articles in this section visit http://onlinelibrary.wiley.com/doi/10.1111/bph.v174.19/issuetoc

**Abbreviations**
ABT: affective bias test
LH: Lister hooded
SD: Sprague Dawley
SPT: sucrose preference test

## Introduction

A number of commonly prescribed, non‐psychotropic medications have been associated with serious neuropsychiatric adverse events such as depression, anxiety and suicidal ideation (Patten and Love, 1994; Celano *et al.,* 2011). While some of these drugs are known to interact with the immune system, such as immunosuppressants and antiviral medications, others spanning a wide range of pharmacological classes have also been observed to increase the risk of these psychiatric side effects; these include anti‐epileptics, cannabinoid receptor 1 (CB_1_ receptor )antagonists/inverse agonists and **nicotinic ACh receptor** partial agonists. Since much of the information regarding these side effects is derived during late stage clinical trials, or from case reports emerging after the drugs have been licensed for treatment (Reith and Edmonds, 2007), many of them have been issued with a black box warning for neuropsychiatric side effects by the Food and Drug Administration (FDA, 2008) or have been withdrawn from the market (FDA, 2009). A major obstacle for drug development is the lack of translational preclinical tests with good predictive validity for pro‐depressant drug effects. The current methods used to assess depression‐like behaviour in non‐human species utilize models of behavioural despair and anhedonia such as the forced swim test, tail suspension test and sucrose preference test (SPT). These methods are predictive of antidepressant efficacy, particularly for drugs acting *via* monoaminergic mechanisms, but these approaches have not provided reliable data for drugs from other classes (Tzavara *et al.,* 2003; Wallace‐Boone *et al.,* 2008). However, recent advances in the use of cognitive neuropsychological testing in human depression research have provided a new opportunity for developing translational methods in laboratory animals. Studies investigating emotion‐induced changes in cognitive processes such as learning, memory and decision‐making have shown that both depressed patients and those with a vulnerability to depression exhibit negative affective biases (Mathews and MacLeod, 2005; Leppänen, 2006; Elliott *et al.,* 2011; Roiser *et al.,* 2012). For example, patients with major depression show reduced recall of positively valenced words (Harmer *et al.,* 2009b), as well as a reduced recognition of happy facial expressions (Joormann and Gotlib, 2006). Importantly, pro‐depressant manipulations have been shown to induce a negative shift in affective processing in otherwise healthy volunteers (Horder *et al.,* 2009). The induction of negative affective bias may therefore represent an early indicator of increased risk for depression, and the use of preclinical assays to detect drug‐induced negative biases may provide an effective early screen for mood‐related side effects.

The development of behavioural tasks to investigate affective biases in animals has progressed significantly in recent years, with studies being conducted in a number of non‐human species (see Hales *et al.,* 2014 for a review). In particular, the affective bias test (ABT) has been used in rats to test for biases in reward learning and memory induced by different affective states (Stuart *et al.,* 2013). Initial validation experiments showed that acute changes in affective state at the time of learning a substrate–reward association biases subsequent choice behaviour during a preference test. It has been demonstrated that a positive affective state, or acute antidepressant treatment, induced a bias towards the substrate–reward pairing learned following the positive manipulation (Stuart *et al.,* 2013; Refsgaard *et al.,* 2016). Conversely, a negative affective state (e.g. induced by psychosocial stress) resulted in a bias away from the substrate–reward pairing learned during the negative manipulation (Stuart *et al.,* 2013).

This present work aims to test the predictive validity of the ABT in detecting acute drug‐induced negative affective biases associated with putative pro‐depressant drugs. Our initial set of studies are designed to test the effects of drugs that interfere with the immune system, by testing two immunomodulators that are used in behavioural research to induce a depression‐like phenotype in animals: the stress hormone corticosterone (Johnson *et al.,* 2006) and the pro‐inflammatory mediator LPS (Kubera *et al.,* 2013). We then tested the effects of IFN‐α, a drug that has been shown to increase the risk for depression and suicidality in patients undergoing immunotherapy (Raison *et al.,* 2005). Following these, we then investigated the effects of a number of drugs with distinct pharmacological mechanisms that have been associated, to varying degrees, with an increased risk of depression in the clinic: tetrabenazine [used as an off‐label treatment for chorea in Huntington's disease (Jankovic and Beach, 1997; Kenney *et al.,* 2006)], varenicline [developed as an aid for smoking cessation (FDA, 2009)], carbamazepine [an anticonvulsant treatment for epilepsy (Mula and Sander, 2007)] and montelukast [a leukotriene receptor antagonist used in the treatment of chronic asthma (Manalai *et al.,* 2009)]. In order to evaluate the impact of long term treatment with pro‐depressant drugs, we also use a modified version of the ABT that allows us to investigate the effects of chronic drug treatments on reward‐induced positive bias. In this version of the assay, animals are trained to associated one substrate with a higher value reward which, during the preference test, induces a bias towards that substrate. We hypothesized that impairments in reward processing, as seen in human depression (see Pizzagalli, 2014 for review), may be reflected as a blunted response in this assay in animals in a putative negative affective state.

## Methods

### Animals

One cohort of 16 male Lister‐hooded (LH) rats (~300–350 g, Harlan, UK) was used to test corticosterone, carbamazepine and varenicline. Two cohorts of 16 male Sprague Dawley (SD) rats (~310–360 g, Charles River, UK) were used in all other studies. Both strains have previously been shown to respond in a similar manner in the ABT when tested using pharmacological and psychosocial manipulations (Stuart *et al.,* 2013: Hinchcliffe *et al*., 2017). Our early work used LH rats, but there is some evidence that the more commonly used SD strain is more sensitive to stress (Deutsch‐Feldman *et al.,* 2015), and so, most of the studies moved to using this strain. The *N* numbers were based on previous studies and power estimates with α = 0.05 and β = 0.8 (Stuart *et al.,* 2013). All animals were housed in pairs under temperature‐controlled conditions and a 12:12 h light–dark cycle (lights off at 0700 h). They were maintained at approximately 90% of their free‐feeding weight by restricting access to laboratory chow (Purina, UK) to ~18 g per rat per day. Water was provided *ad libitum*. All behavioural testing was carried out between 0900 and 1700 h during the animals' active phase. All procedures received ethical approval from the UK Home Office and were conducted in adherence to the regulations of the 1986 Animals (Scientific Procedures) Act and EU Directive 2010/63/EU. Animal studies are reported in compliance with the ARRIVE guidelines (Kilkenny *et al*., 2010; McGrath and Lilley, 2015).

### The affective bias test

#### General protocol

The rats were habituated to a 40 × 40 cm Perspex test arena and trained to dig in two ceramic bowls (10 cm diameter, 5 cm apart) filled with sawdust to obtain a quantity of food pellets (45 mg rodent tablet, TestDiet; Sandown Scientific, UK). Digging training was complete once each rat was able to find the pellets on 12 consecutive trials within 20 s for each trial. Once trained to dig in sawdust, animals underwent a discrimination session consisting of discrete trials where the animal was placed into the test arena and allowed to approach and explore two bowls: one ‘rewarded’ substrate and one ‘blank’ unrewarded substrate. Once the animal started to dig in one bowl (i.e. directed the nose below the surface of the substrate), the other bowl was removed by the experimenter, the latency to dig recorded and the trial recorded as either correct (rewarded substrate) or incorrect (blank substrate). If the animal failed to dig within 20 s, the trial was recorded as an omission. Animals were run until they completed six consecutive correct trials, within a maximum of 20 trials. One SD rat failed to complete the bowl digging training and was therefore excluded from drug testing.

#### Dose–response studies

**Figure 1:**
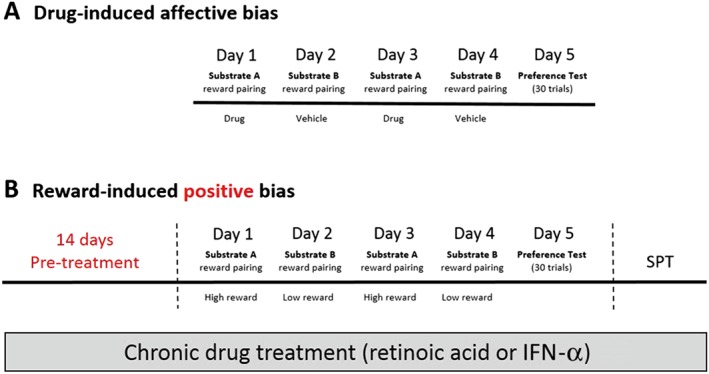
Overview of the ABT protocols. (A) ABT protocol to test drug‐induced affective bias. Animals undergo pairing sessions in the presence of the test drug (e.g. day 1 and 3) versus vehicle treatment (e.g. day 2 and 4). Drug‐induced affective bias is then evaluated in a preference test (day 5) in the absence of drug treatment. (B) Modified ABT protocol to test effects of chronic drug treatment on reward‐induced positive bias. Animals are treated once a day with drug (IFN‐α or retinoic acid) or vehicle for 14 days. Each animal then undergoes pairing sessions where one substrate is paired with a single food pellet reward (e.g. substrate A on day 1 and 3), and the other paired with two food pellets (e.g. substrate B on day 2 and 4). Reward‐induced affective bias is then evaluated in a preference test (day 5) with both substrates randomly reinforced with 1 pellet. Consummatory anhedonia is then assessed in a SPT. Chronic drug treatment is continued throughout the study period.

Each study followed a standard protocol of four pairing sessions followed by a preference test session on the fifth day (Figure 1A). Each pairing session followed the same protocol as the discrimination session described above. All studies used a within‐subject design wherein each animal learnt to associate two different digging substrates (A or B) with a food pellet reward during pairing sessions. These pairing sessions were carried out on separate days following either drug treatment or vehicle. The pairing sessions were carried out on days 1–4 (Figure 1A), and on the fifth day, the rats were presented with both reinforced substrates for the first time and their choices over 30 trials recorded. For the preference test trials, a single pellet was placed in one of the bowls using a random reinforcement protocol such that there was a 1 in 3 probability for each substrate. Trials were run as described above, and an animal's latency to dig and choice of substrate (A or B) was recorded. In all studies, substrate, pairing session and treatment (drug or vehicle) were fully counter‐balanced for each week of the study. The drug doses were administered according to a fully randomised Latin square design with all animals receiving all treatments by the end of the study. Results from the preference test day were recorded as number of choices for the vehicle‐paired substrate versus the number of choices for the drug‐paired substrate and were used to calculate a % choice bias value for further analysis.

#### Chronic drug studies

Animals were treated with drug/ vehicle once daily for 14 days. Each animal then underwent pairing sessions as previously described; however, one substrate (A or B) was paired with a single food pellet reward, and the other paired with two food pellets (see Figure 1B). Preference test trials were run with a single pellet using the random reinforcement protocol. Drug treatment was continued throughout the week of pairing sessions, with treatment being administered at ~1600–1700 h each day, after pairing sessions were complete.

### Sucrose preference test

The protocol for the SPT was adapted from Willner *et al*. (1987). Seventy‐two hours before the first test, rats were given access to one bottle of water and one bottle of 1% sucrose solution in their home cage for 48 h to avoid neophobia during the test. The position of the sucrose solution was counterbalanced across cages and test session. The sucrose solution was then removed and replaced with water until the test day. On test days, animals were deprived of water for 4 h and moved into individual clean cages for 30 min to habituate. Sucrose preference was then determined by a 1 h exposure to two identical bottles filled with either 1% sucrose solution or water. The position of the bottles was counterbalanced across tests using a pseudorandom method. Sucrose preference was defined as the ratio of the volume of sucrose versus water consumed during the test.

### Data and statistical analysis

The data and statistical analysis comply with the recommendations on experimental design and analysis in pharmacology (Curtis *et al*., 2015). All studies were performed with the experimenter blind to drug treatment until the end of the study. Choice bias was calculated based on the number of choices made for the treatment‐paired substrate versus the total number of trials (treatment‐paired substrate + control‐paired substrate). A value of 50 was then subtracted from the choice bias score to give a % choice bias where a bias towards the treatment‐paired substrate gave a positive value and a bias towards the control‐paired substrate gave a negative score. The % choice bias data from dose–response experiments were analysed using a repeated measures ANOVA with DOSE as factor, whilst data from chronic drug studies used an unpaired *t*‐test comparing drug versus vehicle treatment groups. *Post hoc* analysis for each dose/treatment group used a one‐sample *t*‐test against a theoretical mean of 0% choice bias where 0% is equivalent to 15 choices for the treatment‐paired substrate and 15 choices for the control‐paired substrate. Latency and trials to criterion were recorded during pairing sessions to determine whether there were any nonspecific effects of treatment, for example, sedation and anorexia. Analysis of these data was made using a paired *t*‐test comparing treatment versus control for the pairing sessions. Sucrose preference data between drug‐treated and vehicle‐treated groups were analysed using an unpaired *t*‐test. *P* < 0.05 was considered to indicate statistical significance. Statistical analyses were performed using SPSS Statistics 23 and graphs created using GraphPad Prism v7.

### Drugs

Carbamazepine, corticosterone, tetrabenazine, rat recombinant IFN‐α, LPS and 13‐cis retinoic acid were purchased from Sigma Aldrich (UK) and varenicline was purchased from Abcam (UK). Montelukast was purchased from the Bristol Royal Infirmary Pharmacy (UK). Carbamazepine, corticosterone, retinoic acid and varenicline were dissolved in a 10% DMSO, 20% cremaphor and 70% saline mix. Tetrabenazine was dissolved in a 20% DMSO and 80% saline mix at pH 2.0 which was then adjusted to pH 5.5 for dosing. IFN‐α and LPS were resuspended in saline and stocks stored at −20 °C until use. Montelukast was dissolved in saline and adjusted to pH 7.4 for dosing. The saline‐based solutions in which the test drugs were dissolved were administered by the same route and pretreatment procedure to provide a matched vehicle condition in each study. All drugs were administered at a dose volume of 1 mL·kg^−1^ i.p. using a modified handling technique to minimize stress (Stuart and Robinson, 2015). With the exception of montelukast which was administered 20 min before the ABT pairing sessions, all other drugs were given 30 min prior to the session. For the dose–response studies, the range of doses and pretreatment times used were based on previous behavioural studies in rodents [corticosterone: 0–30 mg·kg^−1^ (Johnson *et al.,* 2006); IFN‐α: 0–100 U·kg^−1^ (Kentner *et al.,* 2006); tetrabenazine: 0–1 mg·kg^−1^ (Nunes *et al.,* 2013); varenicline: 0–1 mg·kg^−1^ (Rollema *et al.,* 2009); carbamazepine: 0–30 mg·kg^−1^ (Redrobe and Bourin, 1999); and montelukast: 0–3 mg·kg^−1^ (Ihaku *et al.,* 1999)]. The dose range for LPS (0–10 μg·kg^−1^) was chosen based on pilot data showing locomotor impairment at higher doses, so the maximum dose tested was 10 μg·kg^−1^ (Supporting Information Figure S1). For the chronic studies, the dose of IFN‐α (100 U·kg^−1^) was selected as a result of the acute dose–response data as well as screening for overt sickness behaviour (Supporting Information), and the retinoic acid dose (10 mg·kg^−1^) was selected based on results from our previous work in the ABT (Stuart *et al.,* 2013).

### Nomenclature of targets and ligands

Key protein targets and ligands in this article are hyperlinked to corresponding entries in http://www.guidetopharmacology.org, the common portal for data from the IUPHAR/BPS Guide to PHARMACOLOGY (Southan *et al.,* 2016), and are permanently archived in the Concise Guide to PHARMACOLOGY 2015/16 (Alexander *et al.,* 2015a,b).

## Results

### Effects of immunomodulators on affective bias in the ABT

**Figure 2:**
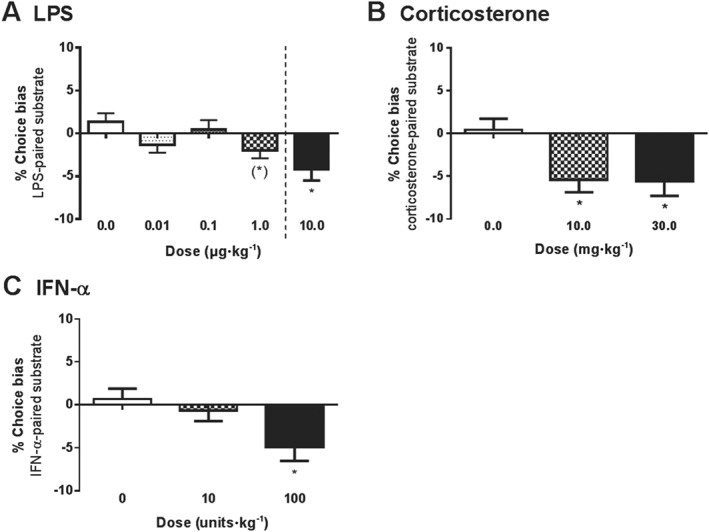
The effect of acute treatment with immunomodulating agents on affective bias in the ABT. Negative biases were observed following acute treatment with (A) LPS (*n* = 15), (B) corticosterone (*n* = 16) and (C) IFN‐α (*n* = 15). Data are shown as the mean % choice bias for the drug‐paired substrate ± SEM, *P < 0.05 (one sample *t*‐test vs. theoretical mean of 0% bias). Dotted line indicates independent experiments where a higher dose was tested following the dose–response study.

In an initial dose–response study with the pro‐inflammatory agent LPS, acute treatment tended to induce a dose‐dependent negative affective bias in rats (*F*_3,42_ = 2.67, *P* = 0.06, *n* = 15; Figure 2A). A single‐dose study was subsequently carried out with a higher concentration of 10 μg·kg^−1^ and was found to induce a significant negative bias (one sample *t*‐test: *t*_14_ = 3.3, *P* < 0.05 vs. 0% choice bias). This dose did not have any effect on latency in the ABT (a food‐motivated task) but did reduce activity in a spontaneous test of locomotor activity (Supporting information). Acute treatment with the stress hormone corticosterone induced a negative affective bias at 10 and 30 mg·kg^−1^ (*F*_2,30_ = 5.52, *P* < 0.05, *n* = 16; Figure 2B). The highest dose also increased the number of trials required to reach criterion in pairing sessions compared to VEH (Supporting Information Table S1). Similarly, acute treatment with IFN‐α induced a negative affective bias at a dose of 100 U·kg^−1^ (*F*_2,28_ = 3.91, *P* < 0.05, *n* = 15; Figure 2C). None of the immunomodulators tested had any effect on omissions during the pairing sessions.

### Effects of putative pro‐depressant drugs on affective bias in the ABT

**Figure 3:**
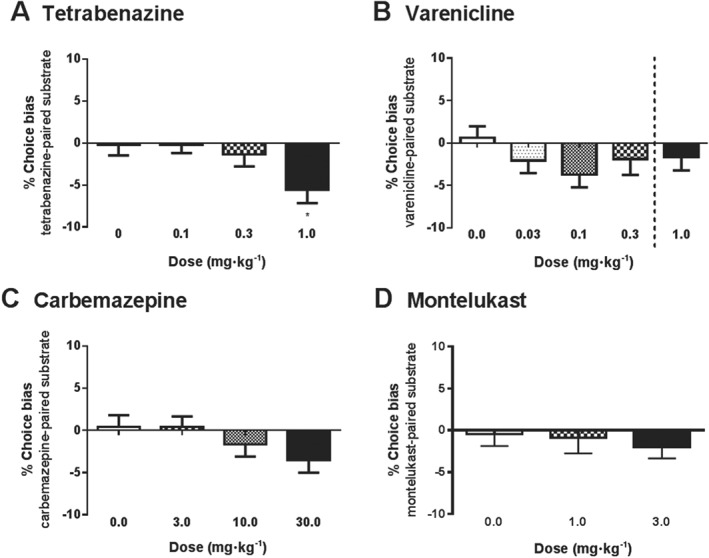
The effects of putative pro‐depressant drugs on affective bias in the ABT. (A) Tetrabenazine (*n* = 15) induced a negative affective bias, but only limited effects were observed with (B) varenicline (*n* = 16) and (C) carbamazepine (*n* = 16). (D) No effect was observed with montelukast (*n* = 15). Data are shown as the mean % choice bias for the drug‐paired substrate ± SEM, *P < 0.05 (one sample *t*‐test vs. theoretical mean of 0% bias). Dotted line indicates independent experiments where a higher dose was tested following the dose–response study.

Tetrabenazine induced a negative affective bias in rats at a dose of 1 mg·kg (*F*_3,42_ = 3.28, *P* < 0.05, *n* = 15; Figure 3A), and whilst varenicline tended to induce a negative bias, the main effect was not statistically significant (Figure 3B). Varenicline was repeated with a single, higher dose of 1 mg·kg^−1^; however, this also failed to induce a significant bias. Carbamazepine tended to induce a negative bias at the higher dose used; however, the effect did not reach statistical significance (*F*_3,45_ = 2.36, *P* = 0.084, *n* = 16; Figure 3C). The test was not repeated with a higher dose (as with varenicline), as animals showed an increase in trial latency during pairing sessions following carbamazepine treatment, suggesting a sedative effect of the drug (Supporting Information Table S1). No overall effect was found with the leukotriene receptor antagonist, montelukast (Figure 3D). None of the drugs tested had any effects on omissions during the pairing sessions.

### Effects of chronic pro‐depressant drug treatment on reward‐induced positive bias and anhedonia

**Figure 4:**
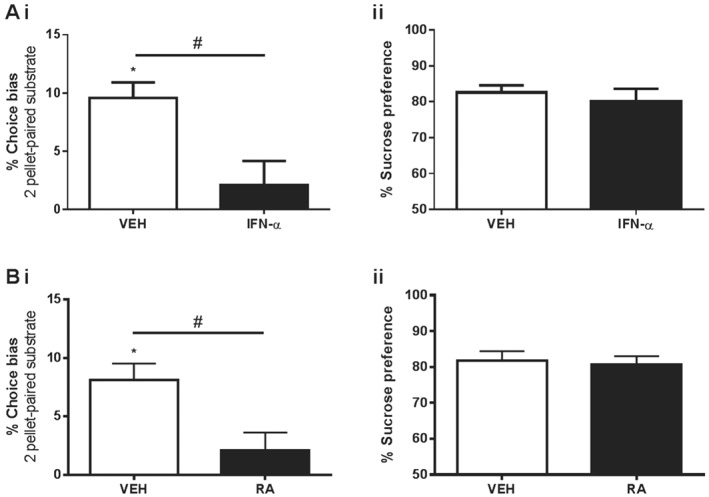
The effect of chronic treatment with putative pro‐depressant drugs on reward‐induced positive bias in the ABT and anhedonia in the SPT. Whilst vehicle (VEH)‐treated animals demonstrated a positive bias for the two‐pellet paired substrate, animals chronically treated with either (A, panel i) IFN‐α (*n* = 8 per group) or (B, panel i) retinoic acid (VEH: *n* = 7, retinoic acid: *n* = 8) failed to show a bias. (A, panel ii, B, panel ii) Sucrose preference was not affected by either drug. Data are shown as mean % ± SEM, *P < 0.05 (one sample *t*‐test vs. theoretical mean of 0% bias), #*P* < 0.05 (independent samples *t*‐test vs. VEH group).

Since IFN‐α was shown to induce a negative bias when administered acutely (Figure 1C), it was then used to investigate the longer‐term effects of chronic treatment on reward‐induced positive bias in the ABT, as well as anhedonia in the SPT. Data from the ABT showed that whilst vehicle‐treated animals demonstrated a positive bias towards the two pellet‐paired substrate (Figure 4A, panel i), this effect was absent in animals treated for 14 days with IFN‐α (unpaired *t*‐test: *t*_14_ = 3.04, *P* < 0.05, *n* = 8 per group). No effect was observed in the SPT, with both treatment groups showing a preference for the sucrose solution (Figure 4A, panel ii). These results were replicated with retinoic acid, a drug that we have previously shown to induce a negative affective bias when given acutely (Stuart *et al.,* 2013). Animals treated for 14 days with retinoic acid show a deficit in reward‐induced positive bias (unpaired *t*‐test: *t*_13_ = 2.84, *P* < 0.05,vehicle: *n* = 7, retinoic acid: *n* = 8; Figure 4B, panel i), but no difference in sucrose preference when compared to control animals (Figure 4B, panel ii). Neither IFN‐α nor retinoic acid affected response latencies, trials to criterion or omissions during pairing sessions (Supporting Information Table S2).

## Discussion

These findings build on our previous work suggesting that the ABT provides a novel translational approach to studying pro‐depressant drug effects and predicting adverse psychiatric side effects in a non‐human model. Consistent with our previous observations (Stuart *et al.,* 2013), drugs which have pro‐depressant effects in patients were observed to induce negative affective biases following acute administration in our ABT. The possible implications for these findings in terms of animal models for psychiatry research and safety pharmacology are discussed below. As well as our studies using acute drug manipulations, we have also found that drugs which induce an acute negative affective bias will also induce deficits in reward‐induced positive bias following chronic treatment. We propose that this deficit may involve similar neurobiological substrates to the anticipatory reward deficit observed in depression (Sherdell *et al.,* 2012) and discuss the implications for both depression research and safety evaluation.

### Effects of immunomodulators on negative affective biases in the ABT

Our initial studies aimed to test whether an acute effect of immunomodulation in the ABT is predictive of the risk for depressive symptoms associated with chronic illness and long‐term immunotherapy. Administration of the pro‐inflammatory endotoxin, LPS is often used as a model of cytokine‐induced sickness behaviour, inducing depression‐like behaviours in animals that are comparable to those observed in chronic stress (Yirmiya, 1996; Kubera *et al.,* 2013). Similarly, high doses of the stress hormone corticosterone have been shown to reliably increase immobility in the forced swim test and reduce sucrose consumption in the SPT (Gregus *et al.,* 2005; Johnson *et al.,* 2006). We observed that when administered acutely, both LPS and corticosterone induced a negative affective bias in rats. It should be noted that the dose of LPS that was effective in the ABT also reduced spontaneous locomotor activity, although it did not have any effects on choice latency during the ABT, suggesting that the measures of motor function in the two assays are not the same. The highest dose of corticosterone increased the number of trials required in the pairing sessions; nevertheless, all animals were able to reach the same criterion of correct choices within the maximum allowed trials. This suggests that the corticosterone‐induced negative bias is unlikely to be a result of a memory deficit, since the animals are still able to form the appropriate substrate–reward association. We also showed that acute treatment with IFN‐α, a drug that is well‐documented to induce depressive symptoms in 20–50% of patients (Raison *et al.,* 2005), induced a similar negative bias which was also dose‐dependent. We observed that animals formed a negative bias away from the experience learned following drug treatment relative to the experience learned under control conditions, suggesting that the relative value of the experience has been reduced, resulting in a negative choice bias. Together, our findings are consistent with our prediction that risk factors for depression such as immune stress and medications that modulate the immune system, share similar effects on reward‐related learning and memory in the ABT.

The development of major depressive disorders in physically ill patients with no previous history of mental disorders is well documented (for review, see Evans *et al.,* 2005), and some of the mechanisms that might be responsible for inflammation‐mediated sickness and depression have now been elucidated (for review, see Dantzer *et al.,* 2008). Chronic activation of the immune system, including immunotherapy, causes a profound reduction in circulating levels of the 5‐HT precursor, tryptophan, which correlates with patients' depression scores (Capuron *et al.,* 2002, 2003). Interestingly, tryptophan depletion has been found to induce a negative bias in the processing of affective information in healthy volunteers (Murphy *et al.,* 2002; Evers *et al.,* 2006; van der Veen *et al.,* 2007). It is therefore possible that the negative biases observed in the ABT following acute immunomodulation may be, at least in part, mediated through changes in the serotonergic system.

### Effects of drugs with purported pro‐depressant risk in clinical populations

Although concerns regarding pro‐depressant effects and increased suicidal ideation and behaviours have been reported for a number of different prescription drugs, empirical data are limited. This has partly arisen due to a lack of valid animal models and the difficulty in demonstrating clear causality when studying clinical populations. In the ABT, we observed a negative bias following treatment with tetrabenazine but no clear effects for the other drugs tested. Depression occurs in up to 15% of patients receiving tetrabenazine treatment for Huntington's disease (Jankovic and Beach, 1997; Kenney *et al.,* 2006), and since it acts as an inhibitor of the vesicular monoamine transporter, VMAT2, it has similar monoamine depleting effects as the known pro‐depressant drug, reserpine (Quinn *et al.,* 1959; Zheng *et al.,* 2006). Neither varenicline, carbamazepine nor montelukast induced a bias in the ABT although at higher doses, both varenicline and carbamazepine did exhibit mean values which were negative. Concerns were raised by the FDA (2009) over the use of varenicline, a nicotinic acetylcholine receptor (α4 subunit) partial agonist, as a smoking cessation aid, due to its association with adverse neuropsychiatric effects. However, its lack of overall effect in the ABT is consistent with a more recent systematic review and meta‐analysis of the effects of varenicline that found no evidence for an increase in depression or suicidal behaviour in patients (Thomas *et al.,* 2015). Similarly, carbamazepine, an anticonvulsant drug, and montelukast, a leukotriene receptor antagonist used for chronic asthma, failed to induce a significant affective bias in the ABT. These results appear to reflect the clinical picture where, despite individual case reports of negative emotion‐related side effects of these drugs, they have not overall been found to increase depressive behaviours in patients (Philip *et al.,* 2009; Andersohn *et al.,* 2010).

An important issue in the field of drug safety research is that the incidence of adverse neuropsychiatric side effects associated with chronic drug treatments are not well defined, and symptoms are seen only in a sub‐population of patients. Therefore, there is a need not only for methods that predict risk of mood‐related side effects but also to understand why certain drugs cause these effects and why only a sub‐population of patients appear to be at risk. Recently, studies suggest that drugs such as tetrabenazine and varenicline may exacerbate depressive symptoms in patients with a pre‐existing psychiatric illness (Kenney *et al.,* 2006; Thomas *et al.,* 2015). Considering the hypothesis that negative affective biases play a key role in the development of persistent negative mood states (Harmer *et al.,* 2009a; Robinson and Roiser, 2016), it is plausible that an increased sensitivity to negative affective bias may underlie individual vulnerability to drug‐induced depression.

### Chronic pro‐depressant drug treatment impairs reward‐induced positive bias, independent of anhedonia in the SPT

Using a modified version of the ABT, we are able to investigate the effects of chronic manipulations on reward‐related learning and memory and their effects on subsequent anticipation of reward. Animals make a decision about which substrate–reward cue to select in the preference test based on their prior experience of the association. We propose that the loss of reward‐induced positive bias reflects a failure to appropriately anticipate the greater value of the substrate paired with the higher value reward during learning. Under normal conditions, rats demonstrate a positive bias towards a substrate that had previously been paired with a higher value of reward. This is consistent with a number of other studies in animal behaviour that have similarly shown that rodents will learn to associate a cue with a higher value reward and subsequently demonstrate a preference for that cue over one that predicts a reward of lower magnitude (Vogel *et al.,* 1968; Lattal and Gleeson, 1990; Giertler *et al.,* 2003). We have been able to show that two drugs that induce negative bias in the ABT following acute treatment, IFN‐α and retinoic acid (Stuart *et al.,* 2013), impaired this reward‐induced positive bias following 14 days of treatment. Interestingly, our data show that this occurs in the absence of a reduction in sucrose preference, replicating findings from other groups that fail to show an effect of chronic IFN‐α and retinoic acid treatment in the SPT (De La Garza *et al.,* 2005; Ferguson *et al.,* 2005). It has been shown previously that sub‐chronic PCP treatment causes an impairment in reward‐induced positive bias in rats, without affecting measures of consummatory anhedonia (Lydall *et al.,* 2010; Sahin *et al.,* 2016). Together with our current data, these findings suggest that the reward deficits observed in the ABT are independent of the anhedonia as measured by the SPT.

While tasks like the SPT are able to quantify consummatory aspects of reward, and how animals experience pleasure at the time of consumption, the deficits in human depression are not associated with a similar impairment (Berlin *et al.,* 1998; Dichter *et al.,* 2010). Depression is more commonly associated with impairments in anticipation of reward, which we propose represent an interaction between cues which predict reward, activation of memory processes and subsequent recall of expected reward value, as well as motivation to obtain the reward. Whilst various methods involving chronic stress have been shown to decrease sucrose preference in rodents (Willner *et al.,* 1992), several researchers have been unable to replicate these findings (Forbes *et al.,* 1996; Harris *et al.,* 1997; Reid *et al.,* 1997). These results from human and animal studies suggest that the SPT is less suitable as an assessment of anhedonia relevant to depressive disorders, and we propose that our current work indicates that reward deficits as measured by the ABT may be more broadly associated with pro‐depressant effects. The ABT requires animals to use their prior experiences of reward‐associated stimuli to guide their subsequent decision behaviour so may be able to tap into the more complex interactions between valuation, prediction and anticipation of reward that play a key role in depression.

## Conclusion

Overall, our data suggest that that ABT shows good predictive validity for pro‐depressant drug effects and allows us to investigate both the acute and chronic effects of drugs on reward‐related affective biases. Our results also suggest that the ABT can be used to model the anticipatory reward deficit observed in depression. Studies designed to investigate the risk of pro‐depressant effects in animals, as a means to predict their effects in humans, are still limited. However, we show that using an objective measure of reward‐related symptoms provides the opportunity to develop animal models with greater translational validity.

## Author contributions

All three authors contributed to the design of the experiments and the preparation of the manuscript. S.A.S. and C.M.W. contributed equally to the collection of experimental data and its analysis. E.S.J.R. obtained funding for the research and managed the programme.

## Conflict of interest

The authors declare no conflicts of interest.

## Declaration of transparency and scientific rigour

This Declaration acknowledges that this paper adheres to the principles for transparent reporting and scientific rigour of preclinical research recommended by funding agencies, publishers and other organisations engaged with supporting research.

## Supporting information


**Figure S1** Effect of LPS (10‐100ug/kg) on locomotor activity in an open field. LPS (10‐100ug/kg) significantly reduced locomotor activity in rats during a 30 min open field test (RM ANOVA: F2,17 = 103.8, *P* < 0.001). Data are displayed as means ± SEM, *n* = 16/group.
**Table S1** Pairing session data from acute studies following drug *vs*. vehicle treatment.
**Table S2** Pairing session data from chronic treatment studies using 2 *vs* 1 pellet protocol.
