# Supplementary material for: Using the affective bias test to predict drug‐induced negative affect: implications for drug safety
Source: Br J Pharmacol. 2017 Aug 30;174(19):3200–10. doi: 10.1111/bph.13972 (PMC5595760; doi:10.1111/bph.13972)
Supplement: Supplementary file 1 — Figure S1 Effect of LPS (10‐100ug/kg) on locomotor activity in an open field. LPS (10‐100ug/kg) significantly reduced locomotor activity in rats during a 30 min open field test (RM ANOVA: F2,17 = 103.8, P < 0.001). Data are displayed as means ± SEM, n = 16/group. Table S1 Pairing session data from acute studies following drug vs. vehicle treatment. Table S2 Pairing session data from chronic treatment studies using 2 vs 1 pellet protocol. [file BPH-174-3200-s001.pdf]

Supporting Information

Locomotor Activity

Ambulatory locomotor activity was assessed in an opaque black Perspex arena (100x100x40cm). Rats were initially habituated to the arena for three 10 minute sessions on consecutive days. On the fourth day, rats received lipopolysaccharide (LPS: 10 or 100ug/kg i.p), or vehicle (0.9% saline) 30 mins before being place in the arena. The animal was tracked for 30 mins using an image subtraction algorithm by Ethovision XT v.10 software (Noldus, US). The distance moved by the animal was recorded in 5-min time bins and analysed using a repeated measures ANOVA.

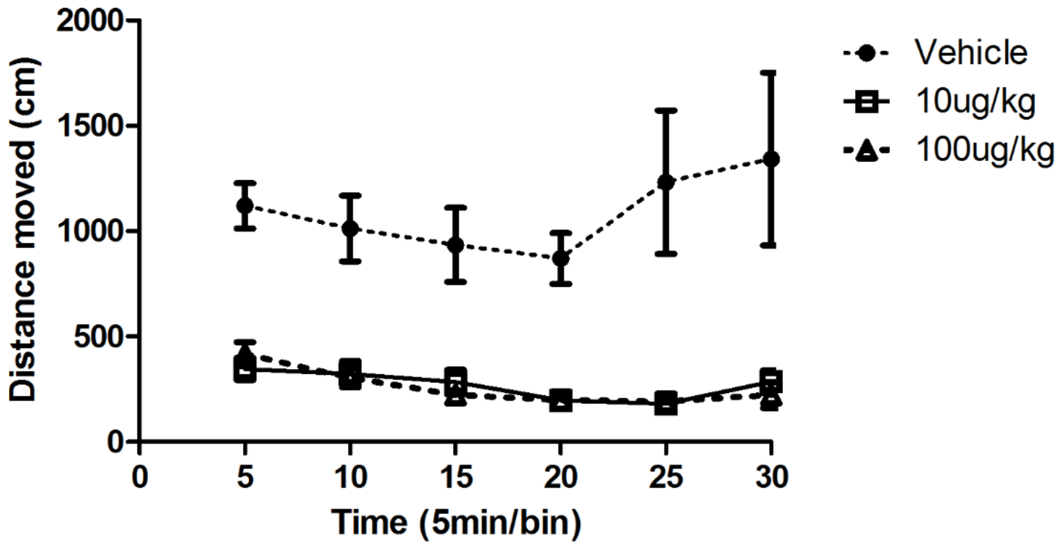

Figure S1. Effect of LPS (10-100ug/kg) on locomotor activity in an open field.

LPS (10-100ug/kg) significantly reduced locomotor activity in rats during a 30 minute open field test (RM ANOVA: $F_{2,17}$= 103.8, p<0.001). Data are displayed as means ± SEM, n=16/group.

Table S1: Pairing session data from acute studies following drug vs. vehicle treatment

Treatment	Dose	Choice Latency (sec)		Trials to criterion		Omissions	
		Drug	Vehicle	Drug	Vehicle	Drug	Vehicle
**Carbamazepine**	0.0	2.7±0.2	2.6±0.2	7.2±0.2	7.3±0.4	0.1±0.1	0.1±0.1
(mg/kg)	3.0	2.5±0.1	2.6±0.1	6.8±0.2	7.1±0.3	0.0±0.0	0.1±0.1
	10.0	2.6±0.2	2.5±0.1	7.1±0.3	7.0±0.2	0.2±0.1	0.0±0.0
	30.0	**2.8±0.2***	2.3±0.1	7.2±0.3	7.1±0.2	0.1±0.1	0.0±0.0
**Corticosterone**	0.0	2.5±0.1	2.6±0.1	7.2±0.4	6.9±0.4	0.0±0.0	0.2±0.1
(mg/kg)	10.0	2.4±0.1	2.5±0.1	7.1±0.3	7.2±0.2	0.2±0.1	0.1±0.1
	30.0	2.3±0.1	2.4±0.1	**7.9±0.5***	6.9±0.3	0.0±0.0	0.2±0.1
**Interferon-α**	0.0	2.3±0.1	2.3±0.1	7.1±0.2	6.7±0.1	0.1±0.1	0.±0.0
(Units/kg)	10.0	2.3±0.0	2.3±0.0	7.1±0.2	7.2±0.2	0.0±0.0	0.0±0.0
	100.0	2.3±0.1	2.4±0.0	6.9±0.2	7.3±0.2	0.0±0.0	0.0±0.0
**Lipopolysaccharide**	0.0	2.6±0.0	2.6±0.0	7.3±0.2	7.1±0.2	0.0±0.0	0.0±0.0
(ug/kg)	0.01	2.6±0.0	2.6±0.0	7.3±0.1	7.2±0.2	0.0±0.0	0.0±0.0
	0.1	2.6±0.0	2.7±0.0	7.4±0.2	7.2±0.2	0.0±0.0	0.0±0.0

	1.0	2.6±0.1	2.7±0.1	7.3±0.2	0.0±0.0	0.0±0.0
	0.0	2.6±0.0	2.6±0.0	7.8±0.3	0.0±0.0	0.0±0.0
	10.0	2.6±0.0	2.6±0.1	8.0±0.2	0.0±0.0	0.0±0.0
**Montelukast**	0.0	2.6±0.1	2.7±0.1	7.9±0.2	0.0±0.0	0.0±0.0
(mg/kg)	1.0	2.6±0.1	2.5±0.1	8.6±0.3	0.0±0.0	0.0±0.0
	3.0	2.9±0.2	2.8±0.2	8.1±0.3	0.0±0.0	0.0±0.0
**Tetrabenazine**	0.0	2.6±0.1	2.7±0.1	6.9±0.2	0.0±0.0	0.0±0.0
(mg/kg)	0.1	2.7±0.1	2.6±0.1	6.8±0.2	0.0±0.0	0.0±0.0
	0.3	2.7±0.1	2.7±0.1	7.1±0.2	0.0±0.0	0.0±0.0
	1.0	2.6±0.0	2.6±0.1	6.7±0.2	0.0±0.0	0.0±0.0
**Varenicline**	0.0	2.9±0.1	2.9±0.1	6.9±0.1	0.0±0.0	0.0±0.0
(mg/kg)	0.03	2.8±0.1	2.5±0.1	7.0±0.2	0.1±0.1	0.1±0.1
	0.1	2.9±0.2	2.7±0.2	7.0±0.2	0.0±0.0	0.0±0.0
	0.3	2.7±0.1	2.8±0.2	6.9±0.2	0.1±0.1	0.0±0.0
	1.0	2.7±0.1	2.5±0.1	7.0±0.2	0.1±0.1	0.1±0.1

Results for response latency, trials to criterion and omissions during pairing sessions following drug vs. vehicle treatment in dose-response studies. Animals were treated with either drug or vehicle 20-30min prior to each pairing session using a fully counter-balanced study design. Data represent the mean value obtained from the two pairing sessions performed under each condition. Carbamazepine increased the animals' response latency at the highest dose, and

corticosterone increased the number of trials to criterion. No significant effects on choice latency or trials to criterion were observed following treatment with the other drugs. Data presented as mean ± s.e.m., n=15-16 animals per group. * $p<0.05$, paired t-test vs. VEH.

Table S2: Pairing session data from chronic treatment studies using 2 vs 1 pellet protocol

Treatment	Dose	Choice Latency (sec)		Trials to criterion		Omissions	
		2 pellet	1 pellet	2 pellet	1 pellet	2 pellet	1 pellet
**Interferon-α**	0.0	2.9±0.1	2.9±0.1	9.3±0.4	8.9±0.6	0.1±0.1	0.0±0.0
(Units/kg)	100.0	2.8±0.1	2.9±0.1	9.4±0.3	9.3±0.7	0.0±0.0	0.0±0.0
**Retinoic Acid**	0.0	2.3±0.1	2.4±0.1	6.9±0.6	8.2±0.6	0.0±0.0	0.0±0.0
(mg/kg)	10.0	2.2±0.1	2.4±0.1	7.8±0.3	7.7±0.4	0.0±0.0	0.0±0.0

Results for response latency, trials to criterion and omissions during pairing sessions following 14 days treatment with IFN-α or retinoic acid. Data represent the mean value obtained from the 2-pellet pairing sessions vs the 1-pellet pairing sessions. No significant effects on choice latency or trials to criterion were observed in drug-treated animals compared to the vehicle group. Data presented as mean ± s.e.m., n=15-16 animals per group.
